# Lost in Translation: Piloting a Novel Framework to Assess the Challenges in Translating Scientific Uncertainty From Empirical Findings to WHO Policy Statements

**DOI:** 10.15171/ijhpm.2017.28

**Published:** 2017-03-01

**Authors:** Tarik Benmarhnia, Jonathan Y. Huang, Catherine M. Jones

**Affiliations:** ^1^Institute for Health and Social Policy, McGill University, Montreal, QC, Canada.; ^2^Department of Family Medicine and Public Health & Scripps Institution of Oceanography, University of California, San Diego, CA, USA.; ^3^Chaire approches communautaires et inégalités de santé, Institut de recherche en santé publique, École de santé publique, Université de Montréal, Montreal, QC, Canada.

**Keywords:** Evidence-Based Policy, Uncertainty, Statistical Variability, Generalizability, Policy Statements, World Health Organization (WHO)

## Abstract

**Background:** Calls for evidence-informed public health policy, with implicit promises of greater program effectiveness, have intensified recently. The methods to produce such policies are not self-evident, requiring a conciliation of values and norms between policy-makers and evidence producers. In particular, the translation of uncertainty from empirical research findings, particularly issues of statistical variability and generalizability, is a persistent challenge because of the incremental nature of research and the iterative cycle of advancing knowledge and implementation. This paper aims to assess how the concept of uncertainty is considered and acknowledged in World Health Organization (WHO) policy recommendations and guidelines.

**Methods:** We selected four WHO policy statements published between 2008-2013 regarding maternal and child nutrient supplementation, infant feeding, heat action plans, and malaria control to represent topics with a spectrum of available evidence bases. Each of these four statements was analyzed using a novel framework to assess the treatment of statistical variability and generalizability.

**Results:** WHO currently provides substantial guidance on addressing statistical variability through GRADE (Grading of Recommendations Assessment, Development, and Evaluation) ratings for precision and consistency in their guideline documents. Accordingly, our analysis showed that policy-informing questions were addressed by systematic reviews and representations of statistical variability (eg, with numeric confidence intervals). In contrast, the presentation of contextual or "background" evidence regarding etiology or disease burden showed little consideration for this variability. Moreover, generalizability or "indirectness" was uniformly neglected, with little explicit consideration of study settings or subgroups.

**Conclusion:** In this paper, we found that non-uniform treatment of statistical variability and generalizability factors that may contribute to uncertainty regarding recommendations were neglected, including the state of evidence informing background questions (prevalence, mechanisms, or burden or distributions of health problems) and little assessment of generalizability, alternate interventions, and additional outcomes not captured by systematic review. These other factors often form a basis for providing policy recommendations, particularly in the absence of a strong evidence base for intervention effects. Consequently, they should also be subject to stringent and systematic evaluation criteria. We suggest that more effort is needed to systematically acknowledge (1) when evidence is missing, conflicting, or equivocal, (2) what normative considerations were also employed, and (3) how additional evidence may be accrued.

## Introduction

The role of research findings and evidence-based public health policy has become of increasing interest to researchers and policy-makers in recent decades.^1,2^ Much of the evidence-based policy research has focused on how to improve evidence utilization and increase uptake, working on a set of assumptions that may neglect consideration of the wider policy processes and decision-making contexts.^3^ Furthermore, there is a lack of knowledge about how uncertainty and conflicting evidence are considered and treated in these processes.^4,5^ This is important because the use of the same evidence for policy can differ according to policy objectives in different decision-making contexts.^6^ Consequently, a large gap exists in understanding and evaluating how policy-making bodies account for uncertainty when using evidence. The World Health Organization’s (WHO’s) role as the health authority in the United Nations (UN) system presents a particularly important institutional context for investigating this issue because its functions include establishing standards and articulating evidence-based policies for implementation in a range of settings and countries around the world. Specifically, its formal mandate includes normative powers for the production of policy recommendations.^7,8^ This paper aims to assess how the concept of uncertainty is considered and acknowledged in WHO policy statements.

### WHO as a Context for Assessing the Translation of Empirical Evidence

As the traditionally recognized international institution that sets norms for public health, there is an expectation for WHO to produce policy recommendations that are based on scientific evidence.^9^ Oxman et al found a general lack of systematic and transparent methods for developing evidence-informed WHO guidelines, and that processes for making recommendations relied on content experts rather than methodologists or guideline users.^10^ Knowledge on the barriers and facilitators of evidence use by decision-makers for health policy appears to support the two communities hypothesis that researchers and policy-makers are two separate groups with distinctly different cultures.^11-13^ However, in the decision-making context of WHO as the international agency responsible for health, the division between these groups is not clear. Commentators have debated about the potential consequences of an overlap in WHO’s technical and political functions in executing its mandate, and the need for transparent processes to ensure scientific credibility and legitimacy with an increasingly crowded landscape of actors involved in global health governance.^14-16^ The use of evidence by WHO has been previously criticized on several fronts.^3,10^ For instance, the diversity of evidence sources used and the transparency of the development process for guidelines has been questioned.^17^ Since the publication of the WHO’s *Handbook for Guideline Development* in 2014, WHO recommendations have been further criticized to be inconsistent with GRADE (Grading of Recommendations Assessment, Development, and Evaluation) guidance.^18^ GRADE is a WHO working group that has developed an approach to grading quality (or certainty) of evidence and strength of recommendations. In general, these critiques focus on the quality and nature of evidence for (or against) the effects of specific interventions. However, as recognized by the 2014 Guidelines, additional sources of evidence are drawn upon to motivate policy recommendations, including “background” evidence. Moreover, the usage of any forms of evidence evaluation (eg, systematic review, GRADE rating), etc nonetheless remain distinct from the final policy recommendations. We propose to evaluate the totality of scientific evidence usage across the policy-making process, paying particular attention to how uncertainty in the scientific evidence base is represented throughout.

### Getting Evidence Into Policy

For evidence to inform policy recommendations, research findings must still be “translated” for decision-makers regarding their applicability for a given setting or population. Knowledge translation is one strategy to improve the use of evidence in policy.^3^ The Canadian Institutes of Health Research (CIHR) define knowledge translation as a “dynamic and iterative process that includes synthesis, dissemination, exchange and ethically-sound application of knowledge” (http://www.cihr-irsc.gc.ca/e/29418.html). Knowledge translation research is a domain of scientific inquiry by itself,^19-22^ producing tools and methods to support implementation of public health knowledge translation activities.^23,24^ However, little attention has been paid to the empirical realities of how evidence “survives,” or may be reshaped, by this translation process. There is a nascent body of knowledge about public health policy-making that demonstrates how evidence is but one kind of argument for influencing policy change in a complex network of policy actors^25^ and how different kinds of research-informed ideas are used by policy-makers to interact within the policy process.^26^ These studies underline the importance of the realities of policy-makers, and policy theory can inform strategies for evidence-based public health policymaking which better account for their decision-making processes, including how they deal with uncertainty.^27^ Given the above, it is problematic that few studies on the use of evidence in policy clearly define the terms evidence, policy or policymaker.^13^

### What Is Scientific Evidence?

In its broadest sense, evidence denotes any “body of facts or information indicating whether a belief or proposition is true or valid.”^28^ Following on from evidence-based medicine,^29^ evidence for public health policy not only refers to general bodies of information, such as demographic or survey data, but also specific products of empirical social and biomedical research, here termed “scientific evidence,” such as results of disease etiology or intervention studies.^2^ It is important to note that there is an ongoing scholarly debate on the definition of evidence for public health policy.^1,30,31^

Brownson et al^32^ propose three categories of scientific evidence relevant for informing public health policy: first, evidence for causal associations between a disease state and a potential risk factor that can be addressed through a public health policy (eg, air pollution and lung cancer^33^); second, evidence of potential impact or effectiveness of a specific intervention (eg, reduction of fuel sulfur content on mortality^34^); third, and least commonly, evidence for “external validity” – what features of and contexts for implementing interventions contributed to the efficiency of the policy. We will refer to these types as Causality, Effectiveness, and External Validity, respectively. Empirical strategies for producing this evidence are diverse and lead to a variety of potential products that may be used to validate truth claims in policy. Among these products, norms are used for classifying and ranking quality, or “strength of evidence” that are widely shared by empirical researchers and embodied by preeminent research institutions.

For example, researchers highly value systematic reviews, exhaustive summaries and syntheses of empirical studies which address a single question or family of questions.^35^ Many guidelines exist to help those who conduct systematic reviews in the service of policy-making. Institutions such as the Cochrane or Campbell Collaborations^36^ provide various tools for conducting systematic reviews and integrating their results in translation processes for policy, especially for evidence of first or second type. In these guidelines, sources of information are generally ranked similarly, with scientific studies subjected to peer-review holding the greatest value, with various criteria about study design and analytic methods creating further subdivisions.^37^ For the purposes of “strength of evidence,” other sources of information are generally considered to be of lesser quality. This includes grey literature or studies not subjected to journal-based peer-review, expert consultation and community-based knowledge.^37^

### What Is Uncertainty?

Uncertainty in the setting of empirical evidence production and translation is understood to arise from various domains. For example, biases in study design and measurement are important source of uncertainty in evidence production. When translating and interpreting empirical evidence, these uncertainties may be represented by specific modalities. Based on the three categories of scientific evidence defined above (Causality, Effectiveness, and External Validity), we have chosen to focus on two modalities: statistical variability and generalizability. Statistical variability can be defined as “the estimated range of values which is likely to include an unknown population parameter, the estimated range being calculated from a given set of sample data.”^38^ It is a way for scientists to systematically demonstrate that they do not know a quantity exactly, for example the proportion of individuals under the age of 45, and the extent to which they are unsure of their estimate. This may be due to several reasons, such as not being able to measure everyone in a target population, and can also be applied to estimates of effect, for example the proportion of individuals that would be cured by a given treatment. In practice, the concept is linked directly to confidence intervals circling a potential ‘true’ parameter. For example, a 95% confidence interval means that if the same population is sampled on many occasions and interval estimates are made at each time, the resulting intervals would circle the ‘true’ population parameter in approximately 95% of the cases. Accordingly, very uncertain estimates, as indicated by wide ranges for confidence intervals, can qualitatively provide an indication that the scientific evidence supporting a certain truth claim (for example, that there is a high proportion of individuals under 45, or that an intervention is effective in curing disease) is weak. Consequently, statistical variability is an important attribute for the first two types of scientific evidence described above – Causality and Effectiveness. In contrast, “generalizability” is related to the extent to which the results of one study can be applied to other contexts and populations and thus directly corresponds to the third type of scientific evidence – External Validity. This notion of external validity has been largely discussed in academic literature, but remains quite a complex topic that defies simple quantification.^39^ Faithfully representing uncertainty that arises from empirical research are fundamental to the process of justifying public health policy recommendations that both (*i*) embrace the variability inherent in empirical evidence-making and (*ii*) consider the issues of applying conclusions from one population/place to another. We note again, that while evaluations of uncertainty (eg*,* bias) of the body of scientific evidence and how it should be used, which is accomplished by many expert bodies including the Cochrane collaboration, the in vivo usage of these evaluations and the source findings themselves in policy statements is a distinct process. To our knowledge, there have been no other evaluations of how these collection of tools ensure, or not, faithful translation of evidence (or evidence synthesis) to actual policy recommendations.

In order to address how these two uncertainty components are translated and incorporated in policy recommendations, we chose to focus on four case studies of WHO policy statements, in which the process of evidence-based policy-making has been given significant attention, and where uncertainty, particularly in generalizing recommendations is an essential consideration.

## Methods

We first reviewed WHO’s standards and rules regarding “evidence-informed” guideline creation to capture the norms and values the organization establishes for this practice. To contextualize our specific policy analyses, we described key features of the WHO guideline development process and the values that motivate them found in the WHO *Handbook for Guideline Development*.^37^ These aspects are presented in Appendix 1.

### Analytical Framework

To our knowledge, this is the first study to-date to investigate the translation elements of uncertainty into public health policy recommendations. Consequently, we propose a novel, practical, and adaptable framework as a first attempt to formalize the evaluation and rating of uncertainty translation (Table). The framework consists of four domains by which we evaluate the translation of each of the two components of uncertainty discussed above (ie, statistical variability and generalizability). The domains are as follows:

**Table:** Analytical Framework for Evaluating Translation of Uncertainty in Empirical Studies to Policy Recommendations

**Domain**	**Criteria**	**Action**
**Statistical Variability**		
Domain A - Use of uncertainty information from empirical work	Reporting values (number of attributable cases, years of life lost, $, % etc)	Count # qualitative (non-numeric) citations Count # point estimates/graphs without error bars Count # point estimates/graphs with error bars
	Reporting associations/risk	Count # qualitative (non-numeric) citations Count # point estimates/graphs without error bars Count # point estimates/graphs with error bars
	Reporting intervention/program evaluation results	Count # qualitative (non-numeric) citations Count # point estimates/graphs without error bars Count # point estimates/graphs with error bars
Domain B - Critique or discussion of empirical work used	Presence of a statement of how they use the original data	Select (Yes/No) and comment(s)
	Acknowledgments of the limits by using original data	Select (Yes/No) and comment(s)
	Discussion about timing of evidence	Select (Yes/No) and comment(s)
	Independent assessment of references	Select (Yes/No) and comment(s)
	Reporting uncited facts	One comment for each instance
	Use/misuse of causal language	One comment for each instance
**Generalizability**		
Domain C - Translation into policy recommendation	Inclusion of a statement or a description about how evidence was used to make WHO recommendations	Select (Yes/No) and comment(s)
	Statement includes qualifications found in citation	Select (Yes/No) and one comment for each instance
	Statement matches finding from citation	Select (Yes/No) and one comment for each instance
	Each recommendation corresponds to findings from one or more citations or systematic reviews	Select (Yes/No) and one comment for each instance
Domain D - Discussion of new uncertainty introduced by the recommendations	Ask for further work in new areas	Propose additional research areas: Comment(s) Discuss criteria for generalizing: Comment(s)
	Introduce limitations of recommendations made	Other health/intervention concerns: Comment(s) New context: Comment(s) Temporal considerations: Comment(s)
	Call for monitoring and evaluation about the recommendations/updates	Select (Yes/No) and comment(s)

Abbreviation: WHO, World Health Organization.

A. Use of uncertainty information from empirical studies,
B. Critique or discussion of such information,
C. Translation into policy recommendation,
D. Discussion of new uncertainty introduced by the recommendation.

For each item, we propose several criteria or subcategories as shown in Table. For Statistical Variability, we consider whether the statement provides representations of statistical uncertainty via presentation of confidence intervals (Domain A) or discussion of the precision and other statistical limitations of cited evidence (Domain B). For Generalizability, we consider how closely recommendations aligned with the interventions evaluated in reviews (C) and whether provide recommendations acknowledged issues with generalizability. These subcategories were organized into a worksheet consisting of both quantitative and qualitative elements (Table). This framework was intended to elicit element we felt to be important to the translation of empirical findings and does not aim to provide a comprehensive list of criteria under all four of the domains that all WHO guidelines or recommendations should be meeting.

Two reviewers (TB and JYH) completed this worksheet for a common documentation in order to test its content. We chose “Closing the Gap in a Generation,” a report of the WHO Commission on Social Determinants of Health,^40^ for the pilot since it was a policy document where the fields of expertise of both reviewers overlapped. Based on this pilot, modifications to the worksheet including eliminating elements for which there was substantial overlap in content and altering commonly described elements, such as confidence interval, from qualitative descriptions to counts. We did not include any ranking of importance for the domains of the analytic framework.

### Case Selection and Materials

Each reviewer then selected two WHO guidelines from those produced between 2008 and 2013 based on their expertise in two fields, namely: Maternal and Child Health and Environmental Health. The four cases of WHO policy recommendations/statements selected were: maternal vitamin D supplementation (JYH); infant feeding (JYH); the heat health action plan (TB); and Malaria Policy Advisory Committee (MPAC) recommendations (TB). While a convenience sample not meant to be representative or systematic, these cases included policy statements from a range of settings in which the evidence base was limited (vitamin D) or extensive (Malaria) or where existing policy was numerous (infant feeding) or rare (Heat).

### Data Analysis

Each reviewer first read the two WHO policy statements corresponding to their field of expertise and described each by summarizing the process of its production, the declared aims of the policy, and its general structure. Then, each reviewer completed a worksheet for each statement. Next, the reviewers summarized general themes and results from their respective worksheets. Finally, the reviewers met to discuss common and emergent themes from the reviews. While each reviewer read the notes and worksheets of the other and discussed interpretations that were unclear, no attempts to cross-verify or replicate the results were made. Again, these reviews were intended to elicit themes regarding the translation of empirical findings and not a definitive evaluation. The reviews were conducted independently between the 2 reviewers. Documents were analyzed using thematic analysis^41^ by combining the four domains and subcategories with additional emergent themes in a deductive way. Each section of the different documents was thus analyzed using this process. Finally, the data was synthesized using identified themes.

## Results

We included four cases of WHO guidelines encompassing different public health policy areas. We found an important heterogeneity across these different guidelines according to the guideline format and structure, the motivations of the document and the overall process. It is also interesting to note that most recently published documents are more transparent in terms of the policy-making process and sources of evidence that are mobilized. This may be due to the publication of different WHO statements in the last years including the *WHO Handbook for Guideline Development* (see details in Appendix 1) in 2014.

### Vitamin D Supplementation in Pregnant Women^42^

This guideline was initiated due to a request from Member States for guidance on “the effects and safety of vitamin D supplementation in pregnant women as a public health strategy” to achieve Millennium Development Goals (pp 1). This interest was informed by beliefs that pregnant women were widely deficient in vitamin D and findings of associations between deficiency and a number of adverse pregnancy and birth related outcomes (Framework Domain B). The guideline specified that Handbook procedures were followed and that evidence was evaluated by a designated existing Guideline Development Group (GDG), the Nutrition Guidance Advisory Group (Framework Domain B) (pp 1). In summarizing the evidence, the GDG identified one Cochrane systematic review on vitamin D supplementation on maternal and neonatal outcomes and made a strong recommendation against supplementation to prevent maternal pre-eclampsia and a conditional recommendation against routine supplementation (Framework Domain D).

Generally, representation of empirical work was qualitative (reporting trends and associations without numbers) when addressing background questions on mechanisms and more precision with numerical estimates and confidence intervals when addressing key (foreground) questions (Framework Domain A). The citation of empirical evidence was fairly evenly split between the two (Framework Domain B). The Cochrane review used to answer key questions was conducted by a WHO epidemiologist of the GDG (Framework Domain C). The summary of evidence, including GRADE criteria, were drawn directly from review and perhaps as a consequence, no further consideration of the strength of the review or any new issues of uncertainty raised was apparent (Framework Domain D). For example, the risk of side-effects from supplementation had a wide confidence interval (risk ratio = 0.17 [95% CI: 0.01-4.06]) and was cited as evidence for no effect, despite substantial uncertainty regarding precision (Framework Domain A) (pp 4). Additionally, questions about the generalizability of the limited randomized controlled trials (RCTs) conducted mainly in France and the United Kingdom or the potential benefits for subgroups, such as vitamin D deficiency, were not clearly addressed (Framework Domain D). Nonetheless, statements regarding key questions were generally measured, confidence intervals were presented to indicate uncertainty, a current lack of high quality studies was acknowledged, and deliberate plans for re-review of evidence in 2016 was stated (Framework Domain D). Moreover, the guidelines were clear to disclose what other factors were considered by the GDG to make recommendations (see Appendix 1). On the other hand, the evidence used to address background questions including questions of potential mechanisms of action, as alluded to above, was less careful in their attempted use of empirical evidence (Framework Domain D). This included the use of uncited facts about biological effects of vitamin D and attribution of casual mechanisms for vitamin D based on theory or single, observational studies (pp 3) (Framework Domain B). Furthermore, a justification of the works cited for background, as the Handbook suggests, or any contrary empirical findings omitted was not apparent.

### Nutritional Interventions Targeted at Young Infants (0–5 months)^43^

These guidelines were adapted from existing guidelines and incorporated into consolidated guidance on “Essential Nutrition Actions: Improving Maternal, Newborn, Infant and Young Child Health and Nutrition.” The guidelines for young infant (0–5 months) feeding evaluated for this paper consisted of a set of four policy recommendations: early initiation of breastfeeding at birth, exclusive breastfeeding for 6 months; mother or donor milk feeding for low birth weight infants; and exclusive breastfeeding by HIV-infected mothers. While there was a stated commitment that most recommendations were updated to comply with the evidence-informed standards in the 2012 Handbook (Preface), it is not clear from the Handbook or the guideline statements how such updating would occur, for example whether new GDGs would be convened to re-asses the evidence (Framework Domain C). Moreover, several of the policy statements were developed in conjunction with other groups such as the United Nations International Children’s Fund (UNICEF), further complicating any adherence to Handbook standards (Framework Domain B).

Overall, statistical variability and confidence intervals were infrequently cited or considered in the presentation of empirical evidence. Similar to the vitamin D guidelines, attempted to use evidence to address background questions as to the importance and burden of child malnutrition through breastfeeding were hampered by uncited facts, and associations cited as causes (pp 2-4) (Framework Domain A). Most notably, the suggestion in the background that there was causal evidence for early breastfeeding in preventing infection-related neonatal mortality cited a single observational study suggesting association (pp 11) (Framework Domain B). Unlike the vitamin D supplementation guideline, however, the lack of consideration for uncertainty was also apparent in the addressing of key questions related directly to the recommended interventions (Framework Domain C). For example, a Cochrane review of community-based interventions to improve maternal and neonatal outcomes was cited as providing a “statistically significant impact on the intuition of breastfeeding within 1 hour of birth” in reference to an average relative risk of 1.94, without any confidence intervals. Additionally, a large confidence interval (7% to 70%) for the effects of early breastfeeding interventions on increasing sustained breastfeeding was considered evidence for being “effective” (pp 14). Relatedly, the generalizability or “indirectness” of summarized evidence was poorly considered, leaving questions of the relevance of systematic reviews to answering key questions as well as the identity of the key questions themselves (PICO-format questions, eg, Population, Intervention, Comparator Interventions, Outcomes, were not apparent) (Framework Domain C). Other evidence cited as relevant systematic reviews include references that were not per se, for example summary of findings from expert consultation (pp 13, Table I-2) (Framework Domain B). Like the supplementation policy, the new uncertainty issues raised by the systematic review were not appropriately considered. Most importantly, while a prominent 2001 systematic review on the impact of exclusive breastfeeding was considered along with a 2009 update, the most recent update in 2012, was not (pp 12) (Framework Domain B). Consequently, subtle issues such as the need to manage infants individually based on growth, or the potential harms of exclusive breastfeeding to iron-deficient infants was overlooked.^44^ Moreover, systematic evidence for certain recommended actions, such as maternal support in the workplace was absent, and other means of justification were unclear (pp 14) (Framework Domain C). On the other hand, the lack of evidence on policies for HIV-infected mothers was clearly and transparently stated and the alternative justifications by principles and context were clearly outlined (pp 17-18) (Framework Domain B). Additionally, the guideline was able to use the identification of sub-optimal study types (non-randomized and cross-sectional) to justify quality of evidence for an equivocal effect of early breastfeeding promotion practices. Finally, the availability of evidence to address the Member State implementation question and special considerations for subpopulations (in the two policies not related to sub-populations) were not considered despite a strong recommendation for implementation in all countries (Framework Domain D).

### The Heat Health Action Plan^45^

This document was motivated by both the extraordinary heat wave that occurred in 2003 in Europe that caused serious health and social issues, and the contemporaneous findings by the Intergovernmental Panel on Climate Change (IPCC) projecting the effects of climate change on human health.^46^ The document’s foreword states that “*Recommendations in this publication are based on results of the two-year project on improving public health responses to extreme weather/heat-waves (EuroHEAT)*.” The stated objective of this report was to “*describe the general principles and core elements of national or regional heat–health action plans, gives options and models for interventions and practical examples and tools from various European countries*.”

The authors of the guidelines did not explicitly present the methodology on which they based their recommendations, but specified that the content was based on research results, experience, and lessons learned (Framework Domain B). They also highlighted that the policy process was mostly based on the EuroHEAT project, which was conducted by an expert panel. This document was also complementary to a technical document “Preparedness and response to heat-waves in Europe, from evidence to action,” which contained their review of the scientific literature. The latter was therefore included in our assessment.

Empirical results (both for associations between heat and health and intervention evaluations) were mostly reported qualitatively (Framework Domain A). Yet in some sections, results and figures, estimates are largely presented with confidence intervals (Framework Domain A). For example, the section about short-term relationships between temperatures and health outcomes highlighted statistical variability, while the section about vulnerable populations did not. Regarding vulnerable populations, it should be further noted that the guideline did not consider some empirical evidence showing opposite results. For example, the statement that “*infants and children are sensitive to the effects of high temperatures*” contrast with other epidemiologic findings including a recent systematic review.^47^

Indeed, similar to the previous policies, the empirical evidence used to motivate background questions, including the etiology of vulnerability to heat, was generally weak (Framework Domain B): A discussion about the determination of the sufficient level of evidence to characterize vulnerability to heat was absent and a single narrative review^48^ was used to describe the phenomenon. Furthermore, neither statistical variability nor generalizability were duly considered when a single study was used to justify the statement (Framework Domain C): “In general, the impact of hot weather on hospital admissions appears to be lower than the impact on mortality.” Moreover, the authors did not include an explicit section acknowledging the limits of available data (Framework Domain B). There is only this brief disclaimer in the foreword: “These suggestions for countries need to be scrutinized for their respective feasibility and applicability on a national or regional basis and may need to be adapted accordingly.” This aspect is not discussed further in the document, nor any motivating evidence provided for guidance.

However, the authors dedicated a large part of the document asking for further study (Framework Domain D), especially policy evaluation studies and providing further etiologic evidence about heat effects and vulnerable subgroups. They also specifically acknowledged that their recommendations only reflect the current, limited quantity of evidence (Framework Domain D). Additionally, authors contributed a dedicated section, including methods and indicators, to support monitoring and evaluation following policy implementation (Framework Domain D).

### Malaria Policy Advisory Committee (2014)

We assessed a set of related recommendations made by the MPAC. This committee was created in 2011 to provide independent strategic advice to WHO on developing policy recommendations on malaria control and elimination. They have a dedicated web page (http://www.who.int/malaria/mpac/en/) which provides a detailed, transparent account of their policy recommendations processes (Framework Domain B). For example, they publish all their expert meeting reports. This committee is supported by evidence review groups and technical expert groups. The former assesses the evidence (both Causality and Effectiveness-types) in the existing scientific literature following the WHO guidelines while the other make the policy recommendations (see description above). Recommendations are made for very specific objectives in malaria control (eg*,* “Intermittent Preventive Treatment [IPT] in Pregnancy,” or “Safety and Efficacy of Gametocytocidal Doses of Primaquine for Plasmodium Falciparum Malaria”) (Framework Domain C).

In evidence review documents, statistical variability is well reported including point estimates and confidence intervals from empirical studies, and discussions thereof (Framework Domain A and B). Critically however, limitations of empirical evidence are generally discussed only in the context of studies that present a challenge to their conclusions or recommendations (Framework Domain B). For example, in the evidence review document on Malaria Diagnosis in Low Transmission Settings, the critique of observational studies is mainly present for negative results about the efficiency of a program.

On their website (see above), they state how they classify the original data they use (Framework Domain B). There, they largely discuss the limited generalizability of evidence from the original data and their recommendations (Framework Domain D). However, a section devoted to discussing the limitations of their own recommendations is uncommon. Additionally, calls for monitoring and evaluation of recommended programs and updates to the evidence are frequently missing (Framework Domain D).

## Discussion

### Summary and Discussion of Findings

First, we must acknowledge that the systematic collection and review of empirical evidence, while the primary fodder for translation evaluated herein, in fact, is only one of many inputs into the WHO policy recommendations development process. Moreover, the practice of adapting existing guidelines, including those developed in conjunction with external partners to adhere to newer standards for evidence-informed policy is not clear or straightforward. Nonetheless, we found the quality of translating uncertainty due to statistical variability and generalizability to be quite predictable with respect to the guidance provided by the Handbook. Specifically, uncertainty was poorly considered in the presentation of evidence used to address background questions of prevalence, mechanisms, and burden or distributions of health problems. Despite guidance to justify any evidence cited,^37^ the use was non-systematic and in many cases, no numerical or confidence interval information was provided, no conflicting evidence presented, and strength of evidence was generally overstated (associations as causal effects). In addition, such poor consideration of uncertainty in the presentation of evidence in WHO guidelines can have important implications. An important implication could be for example that it may not adequately acknowledge the importance of context nor encourage the implementation of local experiments for which implementation and impact evaluation would be conducted. Indeed, if the need for translating and adapting interventions for different contexts is not appropriately acknowledged in a given WHO guideline, it may lead to the direct implementation of an intervention. Yet, by doing so, less evidence will be produced across different contexts that may useful to produce knowledge about generalizability resulting in an unproductive feedback loop.

In contrast, the systematic review of evidence, strongly laid out in the Handbook and supported by numerous known tools and frameworks (PICO, GRADE, Cochrane) was more deliberate in the representation of uncertainty including frequent use of confidence intervals (see details in Appendix 1). However, perhaps due to the limited guidance in the Handbook, little consideration was often given to the generalizability of evidence, alternative interventions, qualifications of systematic review findings, and new issues of uncertainty derived from the reviews. One potential reason is the close working relationships between the GDGs and the reviewers whether they be Cochrane employees, WHO staff, or GDG members themselves. On the other hand, certain subtleties indicated by reviewers, such as potential adverse effects amongst subgroups, were often lost in guideline development in favor of simple recommendations statements. Most troublingly, some recommendations were made without reference to either evidence or any other relevant factors noted by the WHO Handbook (see Table A1 in Appendix 1), thus their appropriateness was difficult to judge. Finally, factors such as publication bias were never considered.

We found the use of evidence, even reviews, to be particularly opaque and deterministic, even when issues of variability are reasonably translated, highlighting the role of evidence as a rhetorical rather than an instrumental informant of policy. Even when evidence was systematically reviewed (eg, MPAC), recommendations often avoided subtle issues raised and may even be unrelated to the evidence produced. These discrepancies were clearest when (*a*) commitments to other standards or recommendations were acknowledged and (*b*) when guideline making deviated from the established standards.

We believe greater care should be taken in the translation of uncertainty, particularly since it is motivation for policy-making in the first place. Moreover, we emphasize that any participants in WHO policy-making processes should make clear when other factors, such as previous knowledge and commitments to action circumscribe or influence the current use of knowledge. In the case of vitamin D supplementation for example, strong recommendations were made on the basis of limited evidence, presumably based on principles of minimal harms. However, such justifications should be more openly discussed. Moreover, newer fields of empirical data analysis have suggested avenues for dealing with statistical uncertainty including estimating bounds for causal effects^49^ or to implement more complex simulation based approaches such as agent-based modelling.^50^ While the application of such practices to policy-making *in vivo* has yet to be explored, at the very least, advisory groups would be served by remembering to include confidence bounds and considerations for other sources of uncertainty. Moreover, while a variety of evidence is indeed needed to motivate new policies (ie*,* in background sections of these documents), better care should be taken with regards to the interpretation of observational evidence including the consideration of contrary findings or alternative interpretations.

We also found some practices to be exemplary, including the MPAC, and further policy recommendations working groups should replicate their efforts at transparency. Yet, one may argue that in many public health fields, the presence of strong empirical evidence such as experimental studies is not as readily available as they were for malaria control. It is often the case that policy is recommended in the absence of strong scientific evidence, and that a recognized need from constituents can be a strong and legitimate sufficient motivation. In these cases, it is especially important to be clear when recommendations do not strictly come from strong scientific evidence. Another good example of this was the feeding recommendation for HIV-infected mothers.

## Limitations

We note again that this study aimed to apply a novel framework to elicit challenges in the translation of uncertainty from empirical studies to policy recommendation. As such, it was not intended, at this stage, to be as a definitive tool for evaluating WHO or other policy statements. Several limitations must be addressed in order for this tool to become more applicable. First, the domains selected for evaluating evidence transition must continue to be refined for both content and reproducibility. Notably, Domain A which is highly quantitative is likely to be highly reproducible while Domain C, which is qualitative and unstructured, unlikely to be so. Additionally, if the framework is itself to be turned in to a generalizable instrument its replicability needs to be tested by independent reviewers (on the same policy statement) and in other policy contexts. Nonetheless, we found this framework to have good-face validity in capturing many of the concerns we had in the use of evidence in the reviewed statements.

## Recommendations

Based on our admittedly preliminary analysis, the notion of uncertainty does not appear to be fully and homogeneously addressed in the four cases of WHO policy recommendations we reviewed. Acknowledging that each public health topic has its own challenges towards the two uncertainty components we addressed in this paper and WHO translation through the policy-making process, we fully recognize the limitations of the framework to completely solve such challenges. However, we encourage policy actors with a strong commitment to evidence-based decision making to consider some of the issues we have explored here, with a particular focus on transparency and systematically including sections of guidelines relating to uncertainty. To that end, we propose the following list of sections to be included in WHO policy recommendations and guidelines:

- Description of how the empirical scientific evidence was assembled and assessed (Causality and Effectiveness).
- Description of how a hierarchy in the empirical data was considered and what approach was used (Framework Domains A, B, C).
- Discussion of the limitations that are presented in the original data. These discussions can include timing of evidence, validity limitations described in the original material, limitations about external validity (Framework Domain B).
- Discussion of the new uncertainty introduced by the policy recommendations. This section can include discussion about generalizability, changing over time, and local policy contexts (Framework Domain D).
- Recommendations and explicit guidelines about monitoring and evaluation of the recommendations introduced (Framework Domain D).

Additionally, we noted that the role of evidence in informing generalizability was largely avoided in the WHO policy guidelines we reviewed. While this may be a function of WHO’s position as needing to inform broad recommendations for diverse member states, it is our opinion that some guidance should be provided to direct how evidence may be used to inform context-specific recommendations and may be needed even in overarching recommendations. One idea may be to employ novel innovations in causal modeling such as generating effect bounds or agent based modelling to operationalize member-state specific knowledge about compliance or local average effects. The use of qualitative methods to better understand barriers and facilitators for implementing WHO policy recommendations and guidelines in specific contexts are complementary to the methods recommended presently. Approaches to guide where generalizability is or is not informed by scientific evidence may be helpful to better understand potential policy efficacy within specific contexts.

## Conclusion

Policy recommendations made by WHO, the foremost public health organization in the world, carry substantial weight with a variety of decision-makers and practitioners. Our assessment of four WHO policy statements and recommendations found that the presentation of the evidence in these cases lacked a clear and systematic consideration of uncertainty, a problem that is exacerbated by absence of explicit references to the evidence selected and the recommendations made in some instances and the inadequate discussion of new uncertainties generated by such policy recommendation. These findings underscore that even in the institutional context of an intergovernmental organization such as WHO, which has a substantial commitment to evidence-based health policy, the interpretation and consideration of uncertainty is an important problem for the instrumental uses of scientific evidence in policy-making. It is unlikely that WHO policy recommendations in the areas we selected are outliers in their treatment of evidence, and we may expect similar results in WHO recommendations on other issues, in particular those with a dearth of experimental studies. This is why we argue that more work is needed to consider how best to translate uncertainty from empirical studies to policy recommendations, with particular attention given to WHO recommendations which are founded more on normative values or principles rather than empirical bases. Our recommended list of additional sections for inclusion in WHO policy statements, recommendations, or guidelines all relate to increasing the transparency of how evidence is used in these documents. Hawkins and Parkhurst^51^ argue that transparency of decisions about identification and evaluation of appropriate evidence and its use to inform policy is a significant criterion for the good governance of evidence. We suggest that the framework used in this paper for the purpose of analyzing how the concept of uncertainty is interpreted and acknowledged in WHO policy recommendations and guidelines may also be, with more testing, a useful contribution to future revisions to the *WHO Handbook for Guideline Development*.

## Acknowledgments

This work is supported by the Montreal Health Equity Research Consortium (Canadian Institutes of Health Research operating grant #115214) which finances Tarik Benmarhnia and Jonathan Y. Huang postdoctoral awards, although no direct funds were received or allocated for the writing of this paper. Catherine M. Jones is supported by a Vanier Canada Graduate Scholarship from the CIHR, Ottawa, ON, Canada Grant No. CGV127503.

## Ethical issues

Not Applicable.

## Competing interests

Authors declare that they have no competing interests.

## Authors’ contributions

TB and JYH contributed equally to this work by conceiving the design of the research. They took the lead in drafting the manuscript. CMJ contributed to the interpretation of the findings, the manuscript writing and provided critical revisions to the manuscript.

## Authors’ affiliations

^1^Institute for Health and Social Policy, McGill University, Montreal, QC, Canada. ^2^Department of Family Medicine and Public Health & Scripps Institution of Oceanography, University of California, San Diego, CA, USA. ^3^Chaire approches communautaires et inégalités de santé, Institut de recherche en santé publique, École de santé publique, Université de Montréal, Montreal, QC, Canada.

## Key messages

**Implications for policy makers**

- Taking World Health Organization (WHO) policy statements as a well-studied context for evaluating the translation of scientific evidence, we suggest that more effort is needed to acknowledge when evidence is missing, conflicting, or equivocal beyond that addressed by systematic review of intervention effects, including in background knowledge. Consequently, it should be made explicit what normative considerations were employed in lieu of such knowledge, and where relevant knowledge gaps exist.
- We propose some recommendations for the consideration of uncertainty in future WHO policy recommendations documents. These recommendations may help provide to policy-makers a more accurate picture of the state of evidence.

**Implications for public**

The translation of uncertainty from empirical research findings into policy recommendations is a persistent challenge because of the incremental nature of research and the iterative cycle of advancing knowledge and implementation. In this paper, we highlight that even when quality of evidence is systematically evaluated with respect to effects of specific interventions, uncertainty with respect to the state of supporting evidence, including issues of generalizability, may be overlooked. This paper reinforces the notion that evidence used to motivate policy is multifaceted and systematic evaluation is quite challenging.

## Appendix 1. A Summary of the World Health Organization’s

From the outset in its introduction, the *WHO Handbook for Guideline Development* situates the reasons for the organization’s practices for developing guidelines within a context of uncertainty that surrounds the decision-making processes for public health policies.

*
“WHO develops guidelines whenever Member States, WHO country offices, external experts or other stakeholders ask for guidance on a clinical or public health problem or policy area. This generally happens when they are uncertain about what to do or how to choose among a range of potential policies or interventions. Uncertainty can be triggered by a new public health problem or emergency; the uncovering of new evidence; an absence of good-quality evidence (or of any evidence at all); or a change in resource availability or access to services.” “1.2. Why does WHO develop guidelines?” WHO Handbook for Guideline Development* (2nd ed.); pp 1.

WHO processes for developing evidence-based guidelines and policy statements are ideal case studies on uncertainty for several reasons. First, as arguably the most widely recognized and influential international body making evidence-informed health policy guidance, WHO has a commitment to a transparent and systematic use of evidence as outlined in their Handbook for Guideline Development and approved by the Guideline Review Committee (https://www.who.int/kms/guidelines_review_committee/en/). As the quotation above indicates, the presence of uncertainty in public health decision-making due to the nature of the available empirical evidence is one of the drivers for developing for these guidelines. To that end, their developers are tasked with assessing the strength of available evidence, including uncertainty due to analytic issues such as study design and statistical variability, using methodologies such as GRADE and evidence from systematic reviews (Figure A1), including performing such work themselves. Second, WHO produces policy guidance for a large number of Member States, and therefore the organization must determine the generalizability of research findings to diverse settings and/ or describe where and why uncertainties regarding translatability will need to be addressed. Third, while “WHO’s legitimacy and technical authority lie in its rigorous adherence to the systematic use of evidence as the basis for all policies,” as alluded to above, the outlined policy development practices embody an understanding that empirical evidence cannot serve as the sole consideration in decision making (Closing the Gap 2008, pp 42: www.who.int/social_determinants/final_report/en/) and in policies that concern health equity and human rights. For example, end-user values and moral imperatives must also be considered (Closing the Gap 2008). Finally, WHO makes guidelines across a wide range of policy areas with a variety of associated evidence bases. Consequently, WHO policy recommendations and guidelines provide ideal cases to study how empirical tools to account for uncertainty operate in public health policy-making, which elements of scientific evidence assessments are successfully translated, which are absent, and whether elements of uncertainty from the policy-making process are either unaddressed by the available evidence or perhaps created a new, based on the evidence.
 Description of Standard WHO Guideline Development Process
We reviewed the WHO Handbook for Guideline Development (http://apps.who.int/iris/handle/10665/145714) to extract the key features of the WHO guideline development processes, specifically focusing on those we think are most relevant to the synthesis and translation of uncertainty from empirical evidence. We identified four critical features of the WHO guideline development process: the expressed commitment to principles, the constitution of special groups, the collection of evidence, and the evaluation of evidence. A descriptive summary of these features and the values that underpin them follows.

The first important feature is WHO’s stated commitment to a principle-based process. WHO defines guideline statements as “recommendations for clinical practice or public health policy,” allowing users to choose and prioritize “among different interventions or measures having an anticipated positive impact on health and implications for the use of resources” (WHO Handbook 2014, pp 1). In accordance to their commitment to use the best science (see Setting and Rationale above), WHO observes the following principles, amongst others: “process and methods” that “aim to minimize the risk of bias in the recommendations”; develop recommendations “based on a systematic and comprehensive assessment of a policy’s or intervention’s potential benefits and harms”; and “recommendations can be implemented in, and adapted to, local settings and contexts” (pp 2).

The constitution of groups with defined roles is the second notable feature. WHO convenes four groups to execute guideline development: *the steering group* administers the entire process including setting objectives, selecting expert groups, drafting recommendations, and overseeing dissemination; *the guideline development group* (GDG) consists of a multidisciplinary group of external (unpaid) experts assembled to assist in question development, assess and interpret GRADE or other evidence, and formulate recommendations; *the external review group of* varied stakeholders are brought in at various stages of policy development to complement missing perspectives from the GDG, and to specifically provided critiques of missing information and implications for implementation; and the systematic review team are contracted expert groups, for example members of the Cochrane or Campbell Collaborations, tasked to perform or update a systematic review of relevant evidence intended to form the basis of policy development. Further, it should be noted that both the GDG and external review groups are constituted to be composed of not only subject-matter experts, but also end users, representatives for target populations, “experts in assessing evidence and developing guidelines,” and economists or experts in equity, human rights, and gender as needed (WHO Handbook 2014, pp 25-26).

How the evidence is collected comprises the third feature of WHO’s guideline development process. The initial stage of evidence collection following formal process initiation involves the formulation of questions that require empirical evidence to address. WHO further divides this process into background questions, whose answers provide context, and foreground questions, whose answers directly inform policy recommendations (WHO Handbook, pp 77). Background questions include mechanisms, prevalence, and distributional questions. Importantly, WHO recognizes that answers for these questions “may be found in a wide range of informational sources, ranging from basic scientific or pharmacokinetic data from animal studies, to surveillance data on incidence cases, to theoretical frameworks” (pp 78). Nonetheless, evidence to answer these questions “must be based on relevant and object evidence in order to generate a high level of confidence in the results” (pp 78). For example, data on incidence or prevalence “should be duly cited and justified” (pp 78). In contrast, foreground or key questions directly pertaining to intervention or policy recommendations including efficacy, effectiveness, potential harms, feasibility, and acceptability “are the most important ones for guideline development” (pp 79). Consequently, they “usually require a systematic review and assessment of the quality of the evidence” (pp 79). Moreover, WHO recommends formulation of such questions in PICO (Population, Intervention, Comparator Interventions, Outcomes) format, to capture the critical attributes of a considered policy.

To answer key questions in PICO format, WHO requires systematic review, which it defines as “a review of a clearly formulated question that uses systematic and explicit methods to identify, select, and critically appraise relevant research, and to extract and analyze data from the studies that are included in the review” (pp 93). To produce these systematic reviews, WHO relies on literature and database searches as well as an existing relationship with the Cochrane Collaboration, a world-renown leader in systematic reviews. Citing several characteristics (Table A1), WHO envisions a proper systematic review to “reduce the risk of bias and improve the reliability an accuracy of conclusions based on evidence” (pp 93).

**Table A1:** WHO Characteristics of a Systematic Review, from page 93 of WHO Handbook on Guideline Development, 2014

• Specific, objective and clearly focused key questions; • Explicit, transparent and reproducible methods; • Pre-defined eligibility criteria for included studies; • A comprehensive and systematic search for all studies that meet eligibility criteria; • An assessment of the risk of bias of the included studies; • A description and synthesis of the characteristics and findings of the individual studies and of the body of evidence; and • Valid and clearly presented conclusions, with information on their applicability to the key question.

Abbreviation: WHO, World Health Organization.

The last important feature relates to the evaluation of evidence. Upon collection of the evidence for systematic review, evaluations of the quality of evidence are conducted, either by in-house WHO staff, commissioned systematic reviewers, and/or the GDG under the guidance of the steering group, with an acknowledgement that an expert in evidence evaluation must be included. WHO provide general guidance for quality assessment, particularly on the use of the GRADE approach to assess the “extent to which one can be confident that an estimate of the effect of association is correct” or alternatively, “certainty of the evidence” or “confidence in the estimates of effect” (pp 110). Notably, GRADE is used to rate a body of evidence for a particular outcome, and not individual studies, based on a quality rating of High, Moderate, Low, and Very Low (Figure A2). By default, a body of evidence based on RCTs “is rated as being of high quality at the outset” while those based on other studies “as being of low quality” (pp 113). These ratings are then modified based on assessment of five criteria: “limitations in study design and execution; indirectness; imprecision; inconsistency; and publication bias” (pp 113).

Importantly for our purposes, the principles of inconsistency, for example widely varying effect estimates, and imprecision, regarding wide confidence intervals, apply specifically to the concept of uncertainty due to statistical variability. Numerous tips are provided to reviewers on how to judge uncertainty due to wide confidence intervals in evidence. Interestingly, the role of GDGs in applying GRADE to assessing evidence is contrasted from standard systematic reviews, in that GDGs “need to consider the context when making a recommendation” rather than solely on the existing evidence itself (pp 117). Moreover, some general guidance is provided on how to evaluate the applicability of findings to different contexts, otherwise known as generalizability or “indirectness” (the lack of generalizability being indirectness). These include the closeness of the evidence to the PICO characteristics (ie, Is it the same population?) and the lack of evidence specifically comparing desired policy options. Finally, guidance is given on major criteria used to translate evidence into recommendations, including the quality of evidence previously adjudicated, values of users, benefits and harms, and resource implications (pp 123). Taken together with the use of opinions of a variety of stakeholders, it would seem that WHO has established a framework for evaluating the nature of uncertainty in the translation of evidence, particularly with regards to statistic variability and generalizability.
